# Registered trials on novel therapies for myasthenia gravis: a cross-sectional study on ClinicalTrials.gov

**DOI:** 10.1038/s41598-024-52539-w

**Published:** 2024-01-24

**Authors:** Xingyue Li, Jinxin Chen, Youtao Wang, Siwei Zheng, Kun Wan, Xiaodong Liu

**Affiliations:** 1 Department of Neurology, Xiangyang No.1 People’s Hospital, Hubei University of Medicine, Xiangyang, China; 2 Hubei University of Medicine, Shiyan, China; 3 Department of Neurology, Taihe Hospital, Hubei University of Medicine, Shiyan, China

**Keywords:** Neurological disorders, Immunology, Neurology

## Abstract

Novel biologics in MG therapy research is on the rise. This research aimed to investigate the characteristics of registered trials on novel therapies for myasthenia gravis on ClinicalTrials.gov. This cross-sectional study used a descriptive approach to assess the features of the included trials on ClinicalTrials.gov. We found 62 registered trials from 2007 to 2023 on ClinicalTrials.gov. The results showed a yearly rise in the number of registered trials (r = 0.76, p < 0.001). Following 2017, more industry-sponsored trials were conducted (91.5% [43] vs. 60% [9], p = 0.009), fewer results were released (10.6% [5] vs. 60% [9], p = 0.001), and more trials entered phase 3 (67.4% [31] vs. 20% [2], p = 0.001). The most researched novel medications were neonatal Fc receptor inhibitors (51.2% [21]), complement inhibitors (39.0% [16]), and B cell depletors (14.6% [6]). According to the website’s data, the neonatal Fc receptor inhibitors and complement inhibitors were effective in treating myasthenia gravis patients in three trials (NCT03315130, NCT03669588, and NCT00727194). This study provides valuable insights into the profile of registered trials on novel therapies for myasthenia gravis. More clinical studies are needed in the future to prove the value of its application.

## Introduction

Myasthenia gravis (MG) is an autoimmune disease mediated by antibodies and with the participation of complement, in which antibodies bind to acetylcholine receptors or functionally related molecules in the postsynaptic membrane at the neuromuscular junction^1^. The standard therapies (i.e., cholinesterase inhibitors, corticosteroids, immunosuppressive drugs, immunoglobulin, plasma exchange, and thymectomy) are effective for many MG patients, even though the pathogenic processes of MG are still not fully understood^2^. However, there are still some challenges in treating MG. On the one hand, some refractory patients don’t do well with such traditional therapies^3^. Conversely, some individuals stop using these medicines because of their adverse effects^4^. To address this issue, numerous innovative treatments, particularly those utilizing targeted biological agents (such as neonatal Fc receptor inhibitors, complement inhibitors, and B cell depletors), have emerged and shown promise in recent years^3^. As a result, these drugs have been the subject of an increasing number of clinical trials globally^5–7^. However, to our knowledge, the survey about registered trials on novel therapies for MG is limited. This study aimed to conduct a cross-sectional investigation about this on ClinicalTrial.gov.

## Methods

### Study design and setting

This cross-sectional study followed the Strengthening the Reporting of Observational Studies in Epidemiology (STROBE) reporting guideline^8^. The institutional review board’s approval was not required since we conducted this study using publicly available data. Clinicaltrials.gov is the most commonly used clinical trial registration site worldwide, and many studies have used data from this site^9,10^. We included trials registered on ClinicalTrials.gov as study subjects.

### Definition of novel therapies

Compared with conventional treatments of MG, the novel therapies included targeted biological medications (such as neonatal Fc receptor (FcRn) inhibitor, CD20 B cell depleting agent, complement inhibitor), chimeric antigen receptor T-cell immunotherapy (CAR-T), hematogenic stem cell transplant, etc. This definition served as our inclusion standard (Supplementary Table 1).

### Data sources and searches

Two investigators (Jinxin Chen and Youtao Wang) independently searched ClinicalTrials.gov. We used words related to MG without any other restrictions. These terms included “myasthenia gravis”, “Myasthenia Gravis, Ocular”, "Ocular Myasthenia Gravis", "Myasthenia Gravis, Generalized", "Generalized Myasthenia Gravis", "Muscle-Specific Receptor Tyrosine Kinase Myasthenia Gravis", "Muscle Specific Receptor Tyrosine Kinase Myasthenia Gravis", "Muscle-Specific Tyrosine Kinase Antibody Positive Myasthenia Gravis", "Muscle Specific Tyrosine Kinase Antibody Positive Myasthenia Gravis", "MuSK myasthenia gravis", "MuSK MG", "Myasthenia Gravis, MuSK", "Anti-MuSK Myasthenia Gravis", "Anti MuSK Myasthenia Gravis" and "Myasthenia Gravis, Anti-MuSK", "Acetylcholine receptor Myasthenia Gravis", "AchR Myasthenia Gravis", "Low-density lipoprotein receptor-related protein 4 Myasthenia Gravis", "LRP4 Myasthenia Gravis", "Agrin Myasthenia Gravis", "Seronegative Myasthenia Gravis", "Bulbar Myasthenia Gravis", "Respiratory Myasthenia Gravis", "Early-onset generalized Myasthenia Gravis", "Late-onset generalized Myasthenia Gravis". All searches were updated until 5th April 2023. The search strategy is as follows: “myasthenia gravis OR Myasthenia Gravis, Ocular OR Ocular Myasthenia Gravis OR Myasthenia Gravis, Generalized OR Generalized Myasthenia Gravis OR Muscle-Specific Receptor Tyrosine Kinase Myasthenia Gravis OR Muscle Specific Receptor Tyrosine Kinase Myasthenia Gravis OR Muscle-Specific Tyrosine Kinase Antibody Positive Myasthenia Gravis OR Muscle Specific Tyrosine Kinase Antibody Positive Myasthenia Gravis OR MuSK MG OR MuSK Myasthenia Gravis OR Myasthenia Gravis, MuSK OR Anti-MuSK Myasthenia Gravis OR Anti MuSK Myasthenia Gravis OR Myasthenia Gravis, Anti-MuSK OR acetylcholine receptor OR AChR OR low-density lipoprotein receptor-related protein 4 OR LRP4 OR Agrin OR seronegative MG OR bulbar MG OR respiratory MG OR early-onset generalized MG OR late-onset generalized MG”.

### Trial selection

Supplementary Table 1 lists the inclusion/exclusion criteria. As for inclusion criteria, we included trials using targeted immunotherapies or other biological agents. And we included both interventional and observational trials. For exclusion criteria, we excluded non-myasthenia gravis diseases. Second, we excluded studies using only conventional treatments for MG without novel agents. Thirdly, we ruled out other unrelated treatments. Finally, we eliminated duplicated trials (see Supplementary Table 4).

### Data extraction

Two reviewers (Jinxin Chen and Youtao Wang) extracted data from the eligible trials independently. Any disagreement regarding the extraction strategy was resolved through discussions. The studied variables included study type, registered year, enrollment, participant age, sponsor, location, center, clinical phenotype, MG autoantibodies, and novel therapies. Also, we gathered information on interventional trials' randomization, blinding, number of arms, assignment, and phase.

### Statistical analysis

As this study's primary analysis method, we mainly employed descriptive statistics. Given that the Food and Drug Administration (FDA) authorized the first novel biologic agent for MG in 2017^9^, we compared the characteristics of clinical trials using 2017 as a time boundary. Continuous variables were reported as median (interquartile range, IQR). Categorical data were described as frequency and percentage. The Mann–Whitney and chi-square tests were used to examine differences between clinical trial characteristics before and after 2017. In the summary of clinical trial outcome data, we collected some effect sizes, including mean (standard deviation, SD), least square mean difference (95% confidence interval, CI), mean difference (95% CI), net mean difference (95% CI), and odds ratio (95% CI). R software (version 4.2.1) and Free statistical software (version 1.7.1, FreeClinical Medical Technology Co., Ltd, Beijing, China) were utilized for all analyses. The threshold for statistical significance was a two-sided P value of 0.05.

### Ethical standard

The Declaration of Helsinki was followed when conducting the study. We achieved this research utilizing data made available to the public. Therefore, institutional review board permission was not required.

## Results

**Figure 1:**
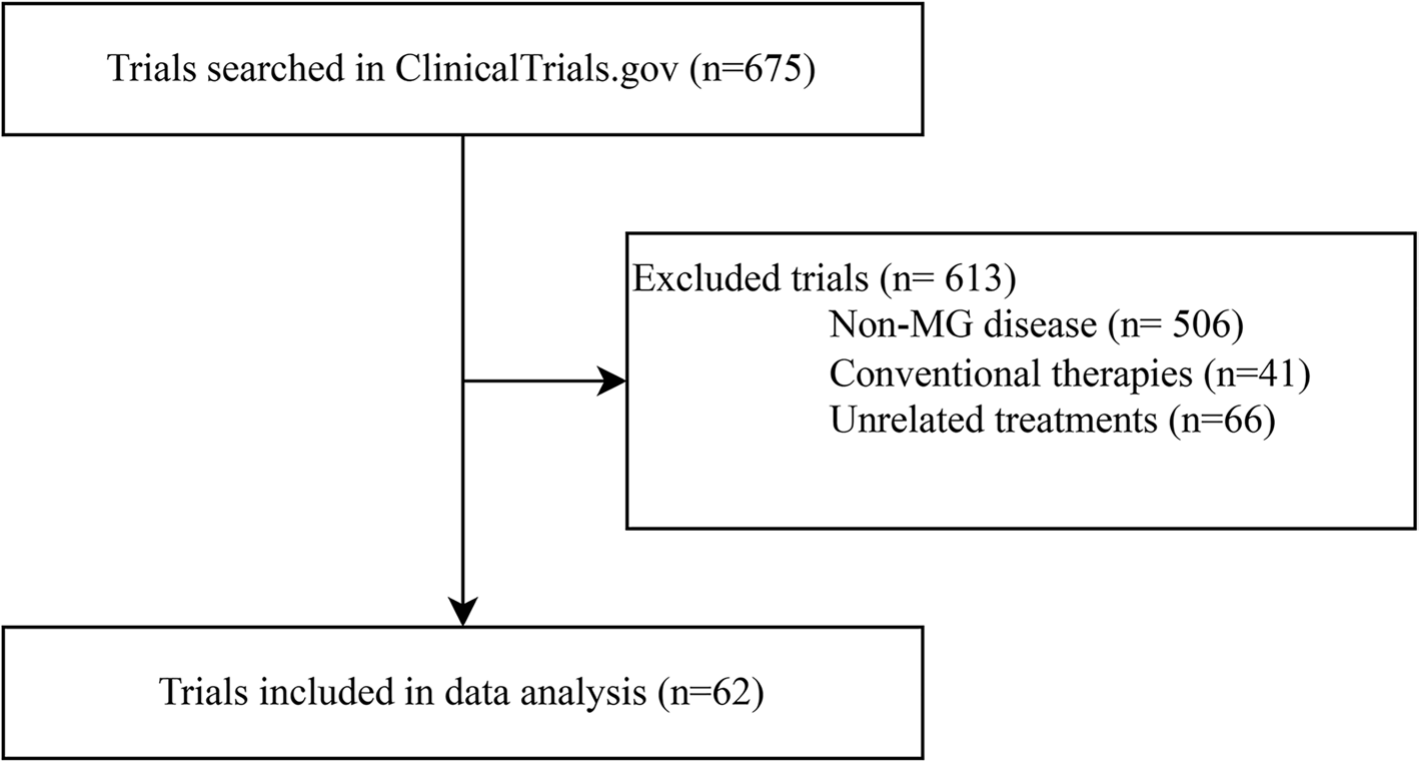
Flowchart of trial selection.

After the initial screening, there were 675 trials on ClinicalTrials.gov in our study. We included 62 studies (registered from 2007 to 2023) for data analysis after discarding 506 trials about non-MG disorders, 41 with only traditional medicines, and 66 unrelated to innovative therapeutics (Fig. 1).

**Figure 2:**
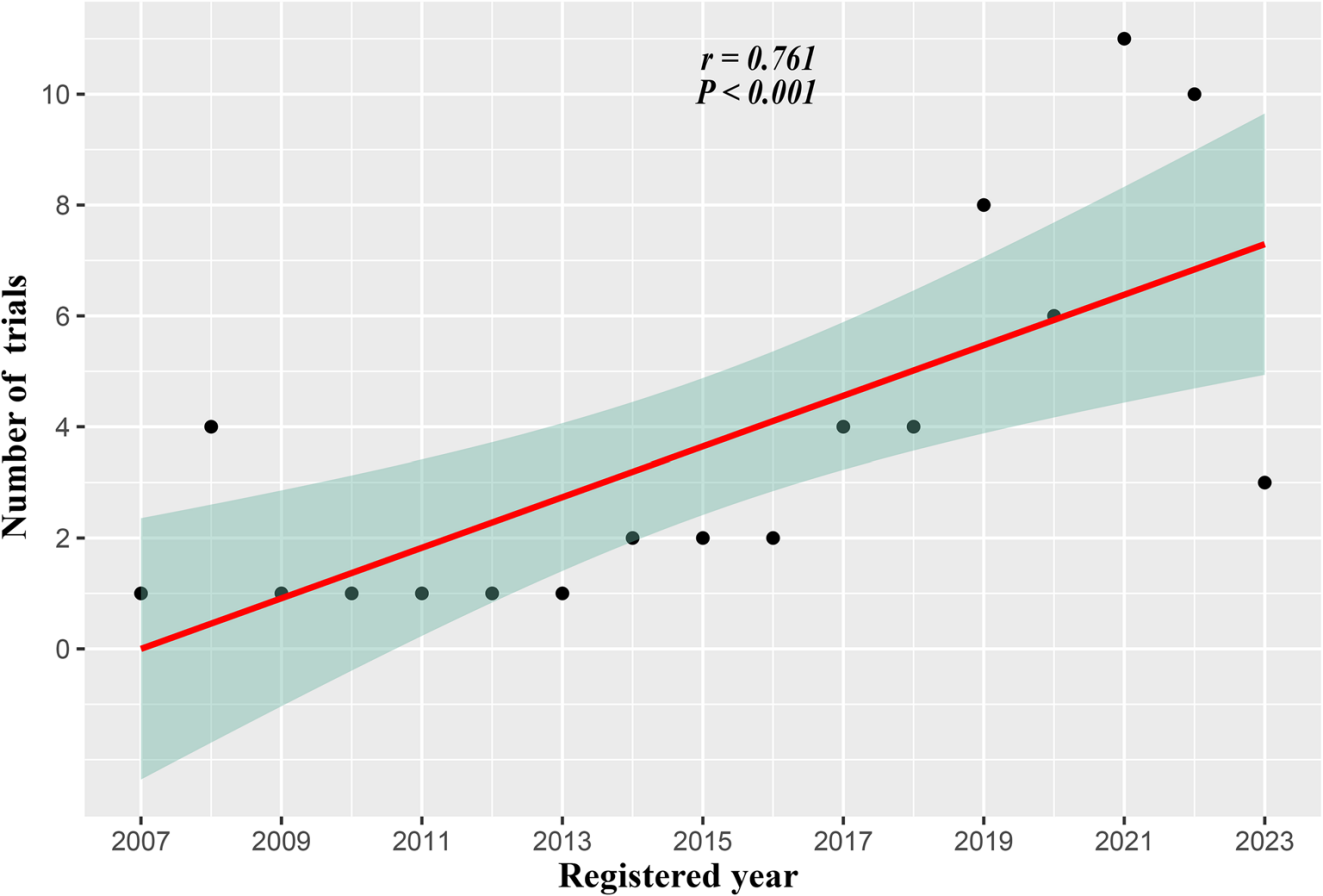
Association between the number of trials and registered year. The solid line and the green region represent the predicted value and 95% confidence intervals, respectively. *CI* confidence interval.

To begin with, we conducted a correlation analysis in Fig. 2 between the number of trials and the year that was registered. This result shows that registered trials increase year-on-year (r = 0.76, p < 0.001). Next, we provided a summary of the trial characteristics in Table 1 (for details, see Supplementary Tables 2 and 3). Following 2017, more industry-sponsored trials were conducted (91.5% vs. 60%, p = 0.009). Second, there were fewer results on ClinicalTrial.gov after 2017 (10.6% vs. 60%, p < 0.001) (for details, see Supplementary Table 3^6,7,11–33^. Moreover, following 2017, more trials entered phase 3 (67.4% vs. 20%, p = 0.001). Other aspects, including research type, participant age, location, center, publication, blinding method, assignment, and randomization, did not alter after 2017.

**Table 1:** Characteristics of eligible trials.

	Total (n = 62)	Before 2017 (N = 15)	After 2017 (N = 47)	*P*-value
Enrollment^a^, Median (IQR)	49 (24, 149)	32 (19, 50)	68 (27, 171)	0.056
Study type, n (%)				1.000
Interventional	61 (98.4)	15 (100.0)	46 (97.9)	
Observational	1 (1.6)	0 (0.0)	1 (2.1)	
Sponsor, n (%)				**0.009**
Industry	52 (83.9)	9 (60.0)	43 (91.5)	
No industry	10 (16.1)	6 (40.0)	4 (8.5)	
Participant age, n (%)				1.000
< 18	3 ( 4.8)	0 (0.0)	3 (6.4)	
≥ 18	52 (83.9)	13 (86.7)	39 (83)	
No restriction	7 (11.3)	2 (13.3)	5 (10.6)	
Location^b^, n^*^ (%)				0.065
Asia	9 (14.8)	0 (0.0)	9 (19.6)	
America	16 (26.2)	6 (40.0)	10 (21.7)	
Europe	6 ( 9.8)	3 (20.0)	3 (6.5)	
Trans-regional	30 (49.2)	6 (40.0)	24 (52.2)	
Center, n^*^ (%)				0.251
Single-center	4 (6.6)	2 (13.3)	2 (4.3)	
Multicenter	57 (93.4)	13 (86.7)	44 (95.7)	
Publication				0.054
No	50 (80.6)	9 (60.0)	41 (87.2)	
Yes	12 (19.4)	6 (40.0)	6 (12.8)	
Results, n (%)				** < 0.001**
No	48 (77.4)	6 (40.0)	42 (89.4)	
Yes	14 (22.6)	9 (60.0)	5 (10.6)	
Blinding				0.597
Blinding	33 (54.1)	9 (60.0)	24 (52.2)	
Open-label	28 (45.9)	6 (40.0)	22 (47.8)	
Assignment^c^, n (%)				0.636
Parallel assignment	36 (59.0)	8 (53.3)	28 (60.9)	
Crossover assignment	4 (6.6)	2 (13.3)	2 (4.3)	
Single group assignment	20 (32.8)	5 (33.3)	15 (32.6)	
Sequential assignment	1 (1.6)	0 (0.0)	1 (2.2)	
Phase				**0.001**
Phase 1/2	27 (44.3)	12 (80.0)	15 (32.6)	
Phase 3	34 (55.7)	3 (20.0)	31 (67.4)	
Randomization, n (%)				0.433
Randomized^*^	39 (95.1)	9 (90.0)	30 (96.8)	
Non-randomized	2 ( 4.9)	1 (10.0)	1 (3.2)	

Significant values are in bold.

*IQR* interquartile range.

^a^Number of patients enrolled.

^b^Participating study sites (country).

^c^ How the intervention was randomly assigned. ^*^Some trials did not provide information about these variables on ClinicalTrials.gov.

Table 2 illustrates the therapeutic effectiveness of the trials on ClinicalTrial.gov. The findings of three clinical trials indicate some advantages regarding novel therapies for MG. Specifically, in a dose–response control trial (NCT03315130), the RA101495 (Complement inhibitor) group's MG scores dropped more significantly than the placebo group at 12 weeks. For the 0.1 mg/kg group, the least square mean difference (80% CI) for myasthenia gravis activities of daily living (MG-ADL) scale, quantitative myasthenia gravis score (QMGS), 15-item myasthenia gravis quality of life revised scale (MG-QoL15r), and myasthenia gravis composite score (MGCS) were − 2.2 (− 3.9 ~ − 0.5), − 2.3 (− 4.5 ~ − 0.1), − 5.3 (− 8.4 ~ − 2.1) and − 2.0 (− 4.9 ~ 0.9), respectively. Corresponding parts for the 0.3 mg/kg group were − 2.3 (− 4.0 ~ − 0.6), − 2.8 (− 5.1 ~ 0.6), − 3.7 (− 6.9 ~ − 0.6), and − 4.1 (− 7.0 ~ − 1.1). Similarly, another trial (NCT03669588) showed that ARGX-113 (efgartigimod, an FcRn inhibitor) significantly reduced the MG-ADL scale compared to the Placebo, regardless of the AChR-ab status. The Odds Ratio (OR) (95% CI) for the AChR-Ab seropositive individuals and the general population were 4.951 (2.213 ~ 11.528) and 3.699 (1.854 ~ 7.578). And the QMGS dropped more in the ARGX-113 group in anti-AChR MG: the OR was 10.842 (4.179 ~ 31.200). In the other crossover-designed trial (NCT00727194), in both periods, the complement inhibitor eculizumab reduced the MG-ADL scale more than the Placebo: the net mean difference (95% CI) was − 1.58 (− 4.08 ~ 0.91). Additionally, eculizumab dramatically decreased QMGS in period one: the net mean difference (95% CI) was − 4.71 (− 10.80 ~ 1.37) (see more in Supplementary Table 5).

**Table 2:** Available results of treatment efficiency of some trials.

NCT number	Year^a^	Action	Intervention	Method of administration	Treatment Intervals	Method of estimation	MG-ADL score^b^		
							Effect Size	*P-*value	Time point (week)
NCT03315130	2017/10/11	Complement inhibitor^c^	RA101495 (Zilucoplan) (0.1 mg/kg) vs. Placebo	SC	Once a day	LS Mean Difference:(80% CI)	− 2.2 (− 3.9 ~ − 0.5)	**0.047**^**d**^	12.00
			RA101495 (0.3 mg/kg) vs. Placebo	SC	Once a day	LS Mean Difference: (80% CI)	− 2.3 (− 4.0 ~ − 0.6)	**0.039**^**d**^	12.00
NCT03052751	2017/5/15	FcRn inhibitor^e^	UCB7665 (Rozanolixizumab) vs. Placebo	SC	Once a week	LS Mean Difference^f^:(95% CI)	− 1.8 (-∞ ~ 0.4)	0.089	4.14
NCT01480596	2013/4	FcRn inhibitor	Belimumab vs. Placebo	IV	Once every 28 days	Mean Difference: ( 95% CI)	− 0.26 (− 2.12 ~ 1.59)	0.775	36.00
NCT00727194	2008/10	Complement inhibitor	Eculizumab vs. Placebo	IV	Once a week to once two weeks	Net Mean Difference(95% CI)	− 1.58 (− 4.08 ~ 0.91)^g^	**0.014**^**d**^	16.00
						Net Mean Difference (95%CI)	− 3.57 (− 6.97 ~ − 0.17)^h^	0.117	16.00
NCT03669588	2018/8/22	FcRn inhibitor	ARGX-113 (Efgartigimod) vs. Placebo	IV	Once a week	OR :(95% CI)	4.951 (2.213 ~ 11.528)^i^	** < 0.001**^**d**^	9.00
						OR(95% CI)	3.699 (1.854 ~ 7.578)^j^	** < 0.001**^**d**^	9.00
NCT01997229	2013/12	Complement inhibitor	Eculizumab vs Placebo	IV	Once a week to once two weeks	Mean Difference (Net): (95%CI)	− 11.7 (− 24.33 ~ 0.96)	0.070	26.00
NCT03896295	2019/8/6	FcRn inhibitor	Placebo-Nipocalimab	IV	Once four weeks	Mean (SD)	− 0.6 (1.14)	NR	36.71
			Nipocalimab-Nipocalimab			Mean (SD)	1.2 (3.08)	NR	36.71
			Nipocalimab (All Participants)			Mean (SD)	0.9 (2.91)	NR	36.71
NCT03772587	2019/4/10	FcRn inhibitor	Placebo	IV	Once two weeks or once four weeks	Mean (SD)	− 2.6 (3.09)	NR	16.14
			Nipocalimab 5 mg/kg			Mean (SD)	− 1.0 (2.25)	NR	16.14
			Nipocalimab 30 mg/kg			Mean (SD)	− 2.8 (2.33)	NR	16.14
			Nipocalimab 60 mg/kg			Mean (SD)	− 2.4 (2.78)	NR	16.14
			Nipocalimab 60 mg/kg (every two weeks)			Mean (SD)	− 2.6 (3.30)	NR	16.14
NCT02565576	2015/9/29	Anti-CD40 monoclonal antibody^k^	CFZ533 (Iscalimab)	IV	Once four weeks	Mean (SD)	− 2.6 (2.97)	NR	25.00
			Placebo			Mean (SD)	− 1.1 (3.23)	NR	25.00
NCT02965573	2016/12/30	FcRn inhibitor	ARGX-113	IV	Once a week	Mean (SD)	− 3.5 (3.50)	NR	11.14
			Placebo			Mean (SD)	− 1.8 (4.22)	NR	78.00
NCT02301624	2014/11/12	Complement inhibitor	Eculizumab/Eculizumab	IV	Once two weeks	Mean (SD)	− 0.7 (4.19)	NR	130.00
			Placebo/Eculizumab			Mean (SD)	− 3.9 (3.68)	NR	130.00

Significant values are in bold.

*NR* not reported, *LS* least-square, *OR* odds ratio, *SD* standard deviation, *CI* confidence interval, *MG-ADL* myasthenia gravis activities of living, *SC* subcutaneous, *IV* intravenous.

^a^Actual Study Start Date.

^b^Evaluation for Change From Baseline over time (The the last follow-up).

^c^These drugs include eculizumab, zilucoplan, etc.

^d^The statistical significance levels were according to the confidence interval.

^e^These drugs include orilanolimab, rozanolixizumab, etc.

^f^1-Sided.

^g^Both Periods.

^h^Period 1.

^i^Analyzed in the AChR-Ab Seropositive Population.

^j^Analyzed in the Overall Population.

^k^These drugs include ravagalimab, iscalimab, etc.

**Figure 3:**
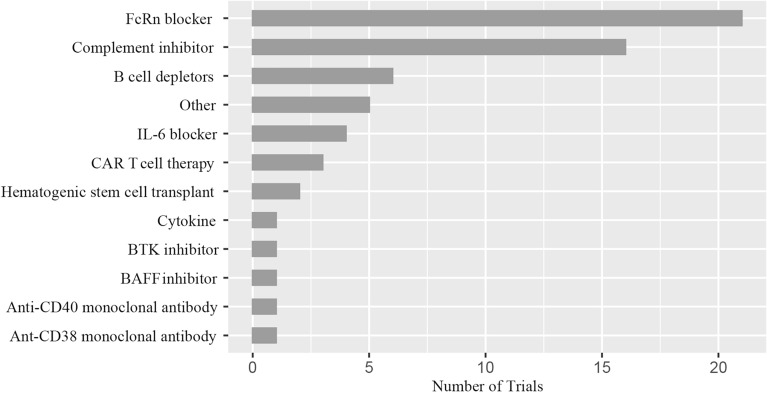
Distribution of novel therapies. *FcRn* the neonatal Fc receptor, *BAFF* B cell-activating factor, *CAR-T* chimeric antigen receptor T-cell immunotherapy, *IL* interleukin, *BTK* Bruton's tyrosine kinase. Other: Orencia (a selective T cell costimulatory immunomodulator); RC18 (TACI-Antibody Fusion Protein), *TACI* transmembrane activator and calcium-modulator and cyclophilin ligand interactor; CK-2017357 (Tirasemtiv, an activator of the fast skeletal muscle troponin complex); CV-MG01 (Myasterix, a kind of vaccine).

Figure 3 compares the number of clinical trials for innovative treatments. The FcRn inhibitor (51.2%) was the most researched medicine on ClinicalTrial.gov. Complement inhibitors (39.0%) and B cell depletors (14.6%) were the second and third, respectively. Other treatments, including IL-6 blockers, CAR T cell therapy, hematopoietic stem cell transplants, cytokines, Bruton's tyrosine kinase (BTK) inhibitors, BAFF inhibitors, anti-CD40 monoclonal antibodies, anti-CD38 monoclonal antibodies haven't been investigated as much.

## Discussion

In this study, we outlined the characteristics of the ClinicalTrials.gov-registered trials testing cutting-edge treatments for MG. Additionally, we looked at the treatment effectiveness data from various trials and discovered some significant results. To our knowledge, the related research on this subject is limited. We believe that a thorough examination of the clinical trials of novel MG treatments could significantly change how we approach clinical practice.

According to our research, there has been an increasing number of registered studies of innovative treatments for MG in recent years. It implies that MG treatment is entering a new age marked by precision medicine^34^. Although many MG patients respond well to traditional therapy, these drugs have certain drawbacks^35^. For instance, clinicians frequently face the challenge of how to treat patients with refractory MG due to poor response or severe adverse effects to conventional medications^36^. As a result, many novel therapies have been applied in clinical research^37^.

Also, the characteristics of these trials indicate that the industry is funding an increasing number of trials. Medicine research is more effective with substantial institutional funding and might ease the transfer from basic testing to clinical use^38^. The pharmaceutical industry supports numerous randomized controlled trials, as they are the primary participants in drug discovery and development^11,13^. On top of that, our investigation identified more clinical registration trials for MG in Phase 3 following 2017. MG's innovative biologics are progressively being utilized in clinical settings^39^. Despite a rise in Phase 3 clinical trials, their outcomes remain comparatively modest for various reasons. They include difficulties in eligible patient recruitment and poor adherence of some patients to protracted follow-up periods^11,18^. Besides, we also found that the percentage of clinical trials with results decreased by 2017. The significant rise in clinical trial registrations could cause this phenomenon. It is well known that any study outcome takes a certain amount of time to complete.

To some extent, it is not surprising that the seemingly contradictory phenomena of fewer results after 2017. Interestingly, no significant difference was found in the relative number of trials published before and after 2017. However, the number of trials published after 2017 (N = 47) was significantly higher than before (N = 15), indicating that there has been a significant increase in the research intensity of novel biologics over the last five years.

The results presented in Table 2 reveal that FcRn and complement inhibitors effectively treat MG. In line with this, Fig. 3's findings also show that they are the most researched medications on ClinicalTrial.gov. And B cell depletor is another novel biologic that has been extensively studied. Following, we covered some elements of these drugs' mechanism of action and clinical research.

Myasthenia is a type of IgG autoantibody-mediated autoimmune disease. IgG recycling is decreased by inhibiting the FcRn receptor as IgG is degraded in lysosomes^40^. Given that IgG production does not compensate for this decrease, FcRn receptor blockade causes a rapid decline in all IgG subclasses^41^. Efgartigimod is a mutated human IgG1 Fc portion with a strong affinity for binding to FcRn^42^. A phase 3 clinical trial^20^ the ADAPT study (NCT03669588) showed that the efgartigimod group had more MG-ADL responders than the placebo group in cycle 1 (2-point improvement in MG-ADL scale lasting for four weeks) (68% vs. 30% p < 0.0001). The OR (95% CI) was 4.95 (2.21 ~ 11.53). Treatment with efgartigimod also resulted in significant and rapid health-related quality-of-life (HRQoL)improvements in generalized MG up to 8 weeks after the first infusion in treatment cycles1(TC1) and TC2^13^. The result shows that FcRn antagonists, represented by efgartigimod, have considerable potential in MG treatment. Notably, efgartigimod has received approval to treat generalized MG globally^43^.

We generally recognize that IgG initiates the complement pathway cascades when it binds to the AChR epitopes. The creation of the C5 convertase marks the culmination of the final steps in this cascade^44^. Eculizumab, a chimeric monoclonal antibody that inhibits the C5 convertase, limits the activation of membrane attack complex (MAC), and reverses the disease status of MG, is one such medication^11^. A global phase 3 clinical trial^11^ (REGAIN (NCT 01,997,229)) revealed that eculizumab performed better than the Placebo in terms of the change in QMGS and MG-QOL15 from baseline to week 26 as determined by worst-rank ANCOVA: the differences (95% CI) were − 16.0 (− 28.5 to − 3.4) and − 14.3 (− 27.0 to − 1.6). It means eculizumab offers long-lasting improvements in patients with refractory generalized anti-AChR MG. A tertiary endpoint analysis^19^ of the REGAIN open-label extension results found that at week 26 of REGAIN, more eculizumab-treated patients than placebo-treated patients achieved a status of improved (60.7% vs. 41.7%) or minimal manifestations (MM) (25.0% vs 13.3%; standard odds ratio: 2.3; 95% confidence interval: 1.1, 4.5). An analysis^23^ which examined changes in the use of immunosuppressive therapy (ISTs) in patients receiving eculizumab during the open-label extension (OLE) of the REGAIN study, found that patients with previously refractory generalized MG used ISTs less frequently (48.7% (57/117)).

Moreover, patients in all groups maintained clinical improvements with eculizumab, including those who decreased or stopped concomitant ISTs. In one subgroup analysis^25^ of REGAIN and its OLE study, the researchers conclude that eculizumab treatment results in meaningful clinical improvements and fewer disease exacerbations for patients who previously received chronic IVIg compared with Placebo. In another interim sub-analysis^27^, eculizumab safety in Japanese and Caucasian patients was comparable to the overall REGAIN population. These results show that eculizumab is of great value in the treatment of MG as a representative of complement inhibitors. Furthermore, the FDA approved eculizumab for treating generalized anti-AChR MG in 2017^11^. Zilucoplan and Ravulizumab are undergoing phase 3 investigations, two drugs with mechanisms of action comparable to eculizumab. These studies align with our findings from Table 2 (NCT00727194).

MG is an antibody-mediated disease and depends on B cells to produce pathogenic antibodies^45^. Thus, the focus of medical research on B cells for MG has garnered attention. Rituximab is a CD20 monoclonal antibody that efficiently depletes most B cells, including memory and immature B lymphocytes^46^. According to a systematic review of case reports on 169 individuals, the number of patients with MG relapse after treatment was significantly reduced in both the anti-AChR MG (93% before vs. 26% after) and the anti-Muscle-specific kinase (MuSK) MG (100% vs. 14%)^47^. Beyond rituximab, other medicines targeted specifically at B-cells have been developed. Obinutuzumab provides a distinct mechanism of action from rituximab through primarily direct cell death rather than complement-mediated cytotoxicity. It may be worth considering as an effective treatment for AChR MG^48^.

Furthermore, ofatumumab, ublituximab, and inebilizumab are also anti-B-cell agents with clinical potential in MG^49^. In one observational study, we observed the efficacy and safety of Inebilizumab (an anti-CD19 monoclonal antibody) in treating of MG (NCT04202341). The website does not display this summary. While these drugs, particularly rituximab, have not yet received marketing approval for MG treatment, we believe that as more relevant clinical trials are conducted, they will soon become valuable tools in treating MG.

Researchers are also testing other innovative therapies, including IL-6 blockers^50,51^, CAR T cell therapy^52^, and BTK inhibitors^53^. An IL-6 receptor inhibitor, satralizumab, prevents IL-6 signaling, which may impact the pathogenic helper T and B cells in MG^54^. It is the subject of an ongoing global phase 3 clinical investigation (NCT 04,963,270). Similarly, Neutrophils, basophils, monocytes, mast cells, neutrophils, and B cells express BTK. It is essential for B cells' activation, growth, and differentiation^55^. Consequently, BTK inhibitors are becoming prospective treatments for MG and other autoimmune diseases^53^. Since there haven't been many clinical registration trials for these drugs, more clinical study is needed to appreciate their potential fully.

There appears to be a greater prevalence of MG today than before. There could have been some reason for this rise in occurrence. For instance, MG used to increase mortality significantly, but over the years, treatment has improved to the point where life expectancy is now almost average in industrialized nations^56^. Furthermore, the increased use of sensitive tests for MG-specific autoantibodies has improved MG case-finding. A recent study from Japan has revealed a natural rise in incidence, especially for late-onset MG^57^. As a result, the therapeutic demand for MG has increased. It is of great significance that many novel and different agents for MG are going on.

On one hand, traditional ISTs, when used over the long term, bring about specific side effects. However, patients can find relative safety in the new biological agents. On the other hand, a notable percentage, ranging from 10 to 30% of individuals living with MG exhibit varying degrees of resistance to conventional immunosuppression due to the severe side effects from therapy or the presence of persistent and incapacitating weakness^1^. Nonetheless, new agents like rituximab emerge as recommended solutions for refractory MG. Uncontrolled studies revealed that rituximab demonstrated effectiveness across all MG groups, displaying varying response rates^47,58,59^. Eculizumab, in patients with refractory AChR, also exhibited a noticeable albeit moderately significant efficacy, as demonstrated in the REGAIN study^18^. Furthermore, even though numerous treatment options are now accessible, the challenge confronting physicians is determining the optimal combination of therapies. This selection hinges on predicting efficacy through an assessment of the clinical phenotype and biological markers of the patients.

There are several limitations to our study. Firstly, the study's cross-sectional nature limited us to further causal analysis. Still, we continue to try to learn more about the traits, particularly the effectiveness, of clinical research on new biologics for MG. Besides, our study's representativeness may have a few drawbacks because it only looked at clinical studies registered on the ClinicalTrial.gov website. Nonetheless, we intend to focus our efforts on other online registries.

Here, it is necessary to reemphasize our findings. To begin with, we have found that the growing number of industry-funded clinical trials is beneficial for translating drug development to the clinic. And this encourages more clinician-scientists and research institutions to engage in various forms of collaboration with businesses. Then, although more clinical trials are moving into phase 3, outputs are still only moderately high. The reason includes difficulties with patient recruitment and poor adherence to extended follow-ups. Therefore, it is crucial to address the problem of successfully grounding clinical trial designs. Finally, our investigation found that the most researched novel biologics are FcRn inhibitors, complement inhibitors, and B-cell scavengers. The result indicates that these medications have great promise for both clinical translation and research utility.

What's more, there are some strengths in our study. First, in contrast to other studies, we statistically analyzed the treatment efficacy of the registered trials and came up with some meaningful findings. Second, we used a comprehensive approach by analyzing the collected trials on both a quantitative and qualitative level.

## Conclusion

In conclusion, this study might offer helpful information on registered studies of cutting-edge treatments for MG. The findings of this analysis would assist clinical researchers or epidemiologists in conducting more high-quality clinical studies. Future evidence-based medicine will also require more well-designed trials.

### Supplementary Information

Supplementary Table 1.

Supplementary Table 2.

Supplementary Table 3.

Supplementary Table 4.

Supplementary Table 5.

## Data Availability

The publicly available datasets for this work are made available online. Online at https://clinicaltrials.gov/, you may find the name of the repository or repositories and their accession numbers.
